# Quantifying evolutionary dynamics from variant-frequency time series

**DOI:** 10.1038/srep32497

**Published:** 2016-09-12

**Authors:** Bhavin S. Khatri

**Affiliations:** 1 The Francis Crick Institute, Mill Hill Laboratory, The Ridgeway, London, NW7 1AA, UK; 2 Division of Infection and Immunity, University College London, London, WC1E 6BT, UK

## Abstract

From Kimura’s neutral theory of protein evolution to Hubbell’s neutral theory of biodiversity, quantifying the relative importance of neutrality versus selection has long been a basic question in evolutionary biology and ecology. With deep sequencing technologies, this question is taking on a new form: given a time-series of the frequency of different variants in a population, what is the likelihood that the observation has arisen due to selection or neutrality? To tackle the 2-variant case, we exploit Fisher’s angular transformation, which despite being discovered by Ronald Fisher a century ago, has remained an intellectual curiosity. We show together with a heuristic approach it provides a simple solution for the transition probability density at short times, including drift, selection and mutation. Our results show under that under strong selection and sufficiently frequent sampling these evolutionary parameters can be accurately determined from simulation data and so they provide a theoretical basis for techniques to detect selection from variant or polymorphism frequency time-series.

Understanding the interplay between stochastic and deterministic forces in systems with different reproducing variants is a theme that arises, and has importance, in many different scientific fields^1^ including language evolution^2^,^3^, protein evolution^4^,^5^, the evolution of biodiversity^6^,^7^,^8^ and population genetics^9^,^10^. In particular, this question has formed the basis of the neutralist-selectionist debate in protein evolution, centred around Kimura’s neutral theory and Hubbell’s neutral theory of biodiversity. With the advent of increasingly sophisticated deep sequencing technologies, the frequency of different variants, or polymorphisms, can now be tracked over time with high resolution. These time-series contain information that could allow very sensitive detection of the relative strength of stochastic drift and selection. Current methods to detect selection, that wholly or partially require the analysis of synonymous versus non-synonymous substitutions, such as the McDonald-Kreitman test^11^ or dN/dS^12^, are not applicable here, since by definition these variants have yet to have fixed in the population and their application can lead to misleading results^13^. Although methods to detect selection from such time-series has attracted much attention recently^14^,^15^,^16^,^17^,^18^,^19^, the bottleneck has been the computational complexity of numerically solving the stochastic dynamics. However, an analytical solution for the stochastic dynamics would enjoy the great advantage of direct evaluation of the likelihood function, allowing very efficient calculation of maximum likelihood parameters or Bayes factors.

To address this goal, we present accurate analytical solutions to a fundamental and long-standing question in population genetics, given the possibility of only two reproducing variants, how does the probability distribution of gene frequency *x*(*t*) change over time, given it is known at a prior time point *x*_0_ = *x*(0), subject to small number fluctuations (genetic or neutral drift), selection (competition) and mutation. We address this question in the context of the Wright-Fisher (WF) model, which is the canonical model of stochastic dynamics incorporating all these features. Although, there have been numerical approaches^15^,^20^ based on series solutions^21^,^22^ of the WF model, these are only valid in the long-time limit where variants will be close to fixation/loss. However, it is of greater practical concern, for example from longitudinal sampling of virus populations^23^, to find solutions valid in the short-time limit, where intermediate changes in polymorphism frequency are observed. The solution of Voronka and Keller^24^, which uses an asymptotic ray approximation, is valid at short times, but their approach lacks simplicity and is unwieldy requiring switching between different solutions in a time-dependent manner.

We present a simple short-time asymptotic calculation of the TPDF in closed form for neutrality, selection and mutation, which has intuitive appeal as it exploits Fisher’s angular transformation^25^. This is the natural co-ordinate for Wright-Fisher stochastic dynamics^26^ and removes the difficulty of a co-ordinate dependent diffusion constant to give simple Brownian motion, at the cost of introducing a non-linear and unstable effective convective force. We show that this force in angular space is directly related to the flux of probability to the fixation and loss boundaries in normal frequency space, which exists despite there being no convection of individual trajectories of the frequency of variants. This is an example of flux without convection, as previously discussed^27^, but in the context of population dynamics. Despite being discussed by Fisher many years ago, Fisher’s angular transformation has not attracted much attention, likely because of the, at first sight, complicated unstable force that arises. We introduce a heuristic approach to overcome this fundamental difficulty, which assumes a Gaussian solution with time-dependent variance calculated from the local derivative of the convective force. We demonstrate that this theory can be used to accurately determine all three evolutionary parameters from simulated data, when under strong selection and sufficiently frequent sampling.

## Results

### Fisher’s angular transformation and the mechanics of neutral *drift*

The diffusion approximation^22^, of the Wright-Fisher model describes the stochastic dynamics of variant frequency *x* (=*n*/*N*, where *n* is the number of copies of a given variant and *N* the total population):


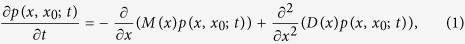


where *p* (*x*, *x*_0_; *t*) is the transition probability density function (TPDF), or Green’s function, of gene frequency given an initial condition *p* (*x*, *x*_0_; 0) = *δ* (*x* − *x*_0_) and *M* (*x*) = *sx* (1 − *x*) + *μ*_1_ (1 − *x*) − *μ*_2_*x* is the mean change in variant frequency per generation, due to selection and mutation and *D* (*x*) = *x* (1 − *x*)/2*N* is half the variance of the variant frequency between generations. This equation is derived, for fixed *N*, in the large *N* limit from a Master equation of discrete populations of each variant^22^. Here *s* is the selection coefficient, where *s* = (*W*_1_ − *W*_0_)/*W*_0_ ≈ *F*_1_ − *F*_0_, so *s* > 0 means selection favours variant 1 over variant 0, where *W* and *F* are the (Wrightian) fitness and (Malthusian) log fitness respectively, and *μ*_1_ is the rate of mutation from variant 0 → 1 and *μ*_2_ the rate for variant 1 → 0.

Fokker-Planck equations with co-ordinate dependent diffusion constants such as Eqn. 1 have the property that space is explored at different rates dependent on the position in the domain; using this intuition, and inspired by the Mahalanobis distance^28^ from statistics, Antonelli *et al*.^26^, suggested the natural definition of length for a stochastic process be related to the differential 
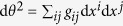
, where *g*_*ij*_ is a metric tensor and taken to be the inverse of the covariance matrix *g*^*ij*^. In one-dimension, this is simply d*θ*^2^ = d*x*^2^/*g*^2^(*x*), which represents the (differential) mean square distance traversed in equal times and *g*^2^ ~ *D* (*x*) the co-ordinate dependent diffusion constant. As the diffusion constant of random drift is *D* (*x*) = *x*(1 − *x*)/2*N*, we choose 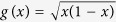, so the natural stochastic distance is simply


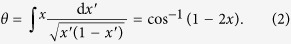


This is Fisher’s angular transformation^25^,^29^.

To examine the underlying mechanics of neutral drift, we focus on the case where there is no selection (*s* = 0) or mutation (*μ*_1_ = *μ*_2_ = 0). Carrying out Fisher’s angular transformation, we arrive at


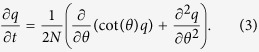


**Figure 1:**
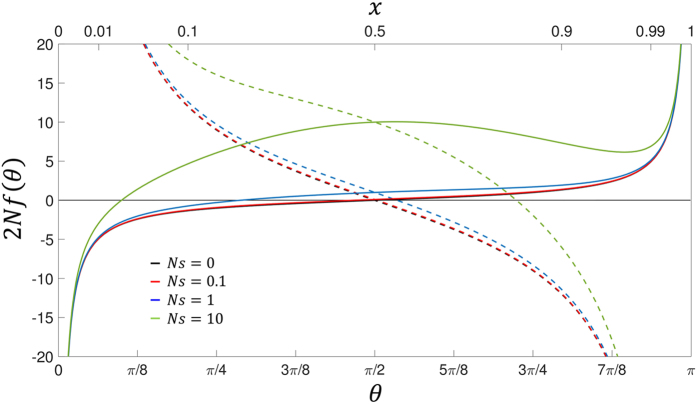
Effective drift force in angular domain for Wright-Fisher process, where 2*N* *f* (*θ*) = (4*Nμ* − 1)cot *θ* + *Ns* sin *θ*, where *μ*_1_ = *μ*_2_ = *μ* compared to Eqn. 5, for 4*Nμ* = 0 (solid lines) and 4*k* = 10 (dashed lines); for 4*Nμ* = 1, drift and mutation exactly balance and 2*N f* (*θ*) = *Ns * sin(*θ*) (not shown). Note that the curves for *Ns* = 0 and *Ns* = 0.1 lie almost on top of each other on the scale of the diagram. We see that the effective force switches from unstable when 4*Nμ* < 1 compared to 4*Nμ* > 1 which is stable, as signified by the change from positive to negative gradient in *f* when it crosses zero.

This is the form of the equation studied by Fisher^25^ for the relaxation of variation in a population. We see that in Fisher’s angular representation, there arises an effective *convective* force, which as shown in Fig. 1 is unstable and so drives diffusers to fixation or loss. Here for clarity, we use the term convection rather than drift, which is the term commonly used in such contexts for Brownian motion. It is simple to show that the origin of this force is the additive term 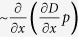 from the original Fokker-Planck equation (Eqn. 1), and is the spurious convective term that arises in transforming between Ito and Stratonovich descriptions of stochastic dynamics^30^,^31^. To understand its significance, we appeal to the continuity equation ∂_*t*_*p* = −∂_*x*_*J* (*x*) (where *J* (*x*) is the probability flux) by integrating Eqn. 1 once with respect to *x* to give 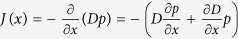. Here the first term is what we would expect by naively replacing the diffusion constant in the standard version of Fick’s law, *J* = −*D*∂_*x*_*p*, with its co-ordinate dependent version, whilst the second term represents an additional or excess flux proportional to the derivative of the diffusion constant, which for neutral drift is −∂_*x*_*D* (*x*) = −(1 − 2*x*)/2*N*. We see that this excess probability flux is exactly of the same form that gives rise to the effective convective potential in Eqn. 3. So this shows explicitly that the co-ordinate dependent diffusion constant of neutral drift gives rise to an excess flux directed towards the boundaries, driving fixation for *x* > 1/2 and loss for *x* < 1/2; in the new co-ordinates this behaviour is manifested by simple (co-ordinate independent) Brownian motion in an effective convective potential. Examining the force in Fig. 1, for the case of *s* = 0 and *μ*_1_ = *μ*_2_ = 0 (black solid curve) we see that it is unstable, on average driving a variant to loss if *θ* (0) < *π*/2 and fixation if *θ* (0) > *π*/2, with a fixed point at *θ* = *π*/2. Note that despite the mean of *θ* having a clear directionality and subject to a convective force, once transformed back to *x*–space 〈*x*(*t*) − *x*(0)〉 = 0, since for asymptotically small times, an Ito stochastic process, such as the Wright-Fisher process, has a differential mean square displacement which is always symmetrical about the initial frequency; this is not true for Stratonovich or isothermal stochastic processes^27^,^30^,^31^. As discussed in detail in ref. 27, it is simple to understand the origin of the excess probability flux to the boundaries; for example, for *x* < 1/2 diffusers travelling from the left have a smaller mean square displacement than diffusers travelling from the right and so there must be a net flux to the left passing an arbitrary point *x* ≠ 1/2, as there is a greater volume of diffusers that reach this point from the right than from the left per unit time. So as is common to mention, the term neutral, or genetic *drift* is a misnomer, as there is no convection/drift of individual trajectories in variant frequencies; however, there is a probability flux of variants moving to the boundaries, suggesting the more appropriate name, *genetic flux*.

Finally, the resulting effective force that arises from Fisher’s angular transformation, as can be seen from Fig. 1 is in general very non-linear, particularly as *θ* → 0 or *θ* → *π*, where the force diverges to infinity towards either the fixation or loss boundaries. This can be understood, since in the frequency domain the diffusion constant tends zero as we approach the boundaries, which means the mean square distances diffused per unit time becomes increasingly small, exactly as given by Fisher’s angular transformation 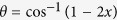; hence, although in the frequency domain the flux is simply linear in *J*(*x*) = −(1 − 2*x*)/2*N* and non-zero at the boundaries, in *θ*-space, linear changes towards each of the boundaries correspond to increasingly small distances in *x* (see the dual-scales in Fig. 1), and so the force must increase as ~1/sin (*θ*) to compensate, in order to maintain a non-zero flux at the boundaries, which is necessary for fixation/loss.

On the other hand, as can be seen from Fig. 1, for frequencies not near the boundaries, the force is linear about *θ* = *π*/2, since the transformation itself is linear in this region; this linearity suggests Gaussian solutions in *θ*-space, and in the Supplementary Online Material we use this fact to calculate very accurate solutions for the TPDF for neutrality (*s* = 0) and *μ*_1_ = *μ*_2_ = 0.

However, the main advance of this paper is presentation of a more general solution, where we present a heuristic methodology which tackles the non-linear effective forces with respect to *θ* due to selection and unequal mutation rates. This general solution therefore has the desirable property of being nestable, as any of the parameters can be set to zero and so is of great practical use in a maximum likelihood analysis.

### Fisher’s angular transformation under selection and mutation

In the presence of selection and mutation, Fisher’s angular transformation results in the following partial differential equation for the TPDF *q*(*θ*, *θ*_0_; *t*):


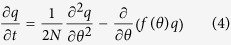


where the effective force is given by,


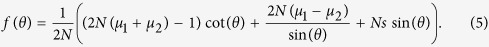


We see that in addition to the effective convective force of neutral drift, there are contributions from 1) mutation with the same *θ* dependence as drift but opposite sign and dependent on the population scaled sum of the mutation rates 2*N*(*μ*_1_ + *μ*_2_), 2) a contribution from mutation, which goes as ~1/sin(*θ*) and proportional to the difference in mutation rates 2*N*(*μ*_1_ − *μ*_2_) and 3) a contribution from selection ~sin(*θ*) proportional to the population scaled selection coefficient *Ns*.

The mutational terms can be understood, since *μ*_1_(1 − *x*) − *μ*_2_*x* = (*μ*_1_ + *μ*_2_)(1 − 2*x*) + (*μ*_1_ − *μ*_2_) and so the first term has the same form as the probability flux due to drift, which as we know transforms to cot(*θ*), whilst the second is a constant force in *x*-space, which means it must diverge as ~1/sin(*θ*) in *θ*-space, as discussed in the previous section due to the particular non-linearity of Fisher’s transformation. The difference in sign, compared to drift, of the first term arises since mutation pushes populations away from the fixation and loss boundaries. Further, the second term is positive if *μ*_1_ > *μ*_2_, as the net tendency will be for a flow of mutations into the variant, rather than the existing ‘wildtype’, whilst if *μ*_1_ < *μ*_2_ this tendency reverses sign.

The contribution of the force due to selection can also be rationalised; this force varies as ~sin(*θ*), which tends to zero as *θ* → {0, *π*}, which agrees with the intuition that when a variant is rare (and there are no mutations), the change in variant frequency is dominated by neutral drift; in particular, for *θ* ≪ 1, and *Ns* ≫ 1, 2*N* *f* (*θ*) ≈ −1/*θ* + *Ns* *θ* and the forces of drift and selection are roughly in balance when *Ns* ~ 1/*θ*^2^ = 1/4*x*, where Fisher’s angular transformation is 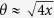 for *x* ≪ 1 – in other words when the variant frequency *x* ≪ (4*Ns*)^−1^ drift dominates. A similar analysis including mutation shows drift dominates for 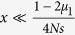 assuming 2*Nμ*_1_ < 1; as 2*Nμ*_1_ approaches 1 from below, the critical frequency at which drift dominates becomes increasingly small. Finally, as is well-known from equilibrium analysis of Wright^32^ and recapitulated here in a dynamical setting in the angular domain, when the strength of mutations switches from weak to strong (2*N*(*μ*_1_ + *μ*_2_) ≫ 1), the force switches from being unstable to stable (as shown in Fig. 1 for *μ*_1_ = *μ*_2_ = *μ*), signifying a transition from the monomorphic regime to the polymorphic.

### Heuristic Gaussian solution

To solve Eqn. 4 and Eqn. 5 approximately, for any value of *N*, *s*, *μ*_1_ and *μ*_2_, we present a heuristic approach that assumes the TPDF can be approximated by a Gaussian process with time-varying mean and variance; where: 1) the time-varying mean is approximated by the solution to the effective deterministic dynamics of the PDE Eq. 4 with initial condition *θ*_0_; and 2) the time-varying variance 〈〈*θ*^2^(*t*)〉〉 = 〈*θ*^2^(*t*)〉 − 〈*θ*(*t*)〉^2^ is dependent on the local gradient of the force, which varies as a function of the solution of the mean, *λ* = *f* ′(〈*θ*〉). The approach here is similar to the work by Feder *et al*.^17^ in their inference of genetic time-series, itself based on earlier works^33^,^34^,^35^, where a deterministic mean is used in a Gaussian approximation of the stochastic dynamics; however, here the key novelty is use of Fisher’s transformation, which first removes the difficulty of co-ordinate dependent diffusion.

It is first most transparent to write Eqn. 4 in its equivalent stochastic differential equation (SDE) form refs 30 and 31:


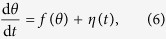


where *f*(*θ*) is given by Eqn. 5 and *η* (*t*) is a Gaussian random variable with zero mean (〈*η* (*t*)〉 = 0), variance 1/*N*, and is uncorrelated with itself except at equal times (〈*η* (*t*) *η* (*t*′)〉 = *δ*(*t* − *t*′)/*N*). The first step is to calculate the time-varying mean of the TPDF in *θ*-space. Although, it is clear that in general for a non-linear SDE such as Eqn. 6, 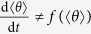 and is, in principle, a function of all moments, we make this approximation, which we will see is very reasonable with respect to calculating accurate solutions for the TPDF. However, to be clear that this is not strictly a solution for the mean and effectively the solution to the deterministic equation, we denote the solution to Eqn. 6 with *η* = 0 as Θ(*t*). Transforming the equation for Θ back to *x*–space, we have a differential equation for 
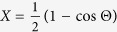



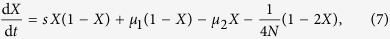


which is just the ODE for the deterministic dynamics of selection and mutation, but including the last term on the RHS which is an effective “deterministic” force due to neutral drift, which we will see is necessary for accurate solutions. The solution to Eqn. 7 is of the form *X* = *C* + *A* tanh (*γt*/2 + *α*). Transforming back to *θ*–space and using the initial condition *θ*_0_ = Θ (0), the solution Θ is:


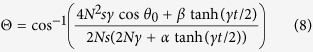


where 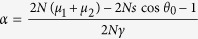, *β* = (2*N*(*μ*_1_ + *μ*_2_) − 1)*α* − 4*N*^2^*γ*^2^, and the characteristic rate of change of Θ is


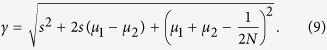


Note that this calculation for Θ only makes sense for short times; for long times when the argument of 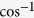 is greater than one, we set Θ = 0 and when the argument is less than −1, we set Θ = *π*; this corresponds to loss and fixation in a deterministic sense, respectively.

The next step is to calculate the variance, which we motivate by considering the situation when the slope of the effective force is fixed to a constant *λ*, which gives a Gaussian solution with variance 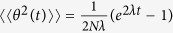 (see Supplementary Online Material). The linearity of the force characterises the Gaussian distribution and so if we assume that the effective convective force varies slowly over a range of theta representing the width of the probability density, we can then heuristically replace *λ* with the local derivative of the effective force *λ*(Θ) in the variance. This approximates the local spreading of the probability density being solely due to the local derivative of the force giving a time varying variance:


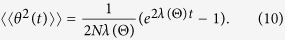


Note that for strong selection, the derivative of the effective convective force *λ*(Θ) will be zero at certain times, as can be seen from the plot of the effective convective force in Fig. 1; at these time points it is simple to see that the variance remains well behaved as 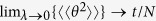, as one would expect if the effective convective force tends to a constant. Transforming back to *x*–space, and using the fact that 
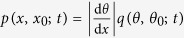
, where the Jacobian is 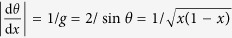 the TPDF solution is:


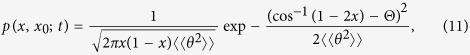


where Θ and 〈〈*θ*^2^〉〉 are given by Eqns 8 and 10, respectively, where cos(*θ*_0_) = 1 − 2*x*_0_. This is the main result of the paper and is a universal and accurate formula for calculating the TPDF of 2-allele population genetics at short times; this result by itself will be useful for many applications including longitudinal sampling of virus populations to study intrahost evolution, since population sizes will be large and selection coefficients typically small, meaning even relatively infrequent sampling can be captured with Eqn. 11 as long as the times between samples is less than *N* or 1/*s*. However, for long times the solution does not strictly obey the boundary conditions at *x* = 0 and *x* = 1, where the solutions develop singularities. The method of images cannot be used to meet the boundary conditions, as in this case as the required images have their forces reversed and so does not obey the original Fokker-Planck equation. In the methods we detail modifications of the theory to give well-behaved results near the boundaries, however, since we do not explicitly consider the nature of the solutions at these singular points, we expect our results will be accurate to *O*(*τ*), where *τ* is the mean time to fixation, which for pure drift is *τ* ~ *N* and including selection 
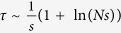
36.

**Figure 2:**
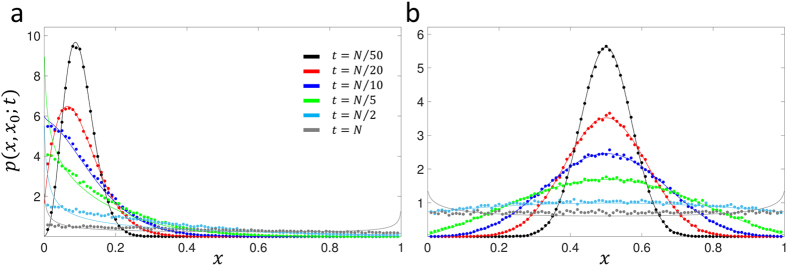
Comparison of approximate calculation of neutral Greens function (*s* = 0, *μ*_1_ = *μ*_2_ = 0) using heuristic Gaussian method (solid lines – Eqn. 11) and numerical integration of stochastic differential equation that arises from diffusion approximation (solid circles). (a) initial frequency *x*_0_ = 0.1, (b) *x*_0_ = 0.5.

**Figure 3:**
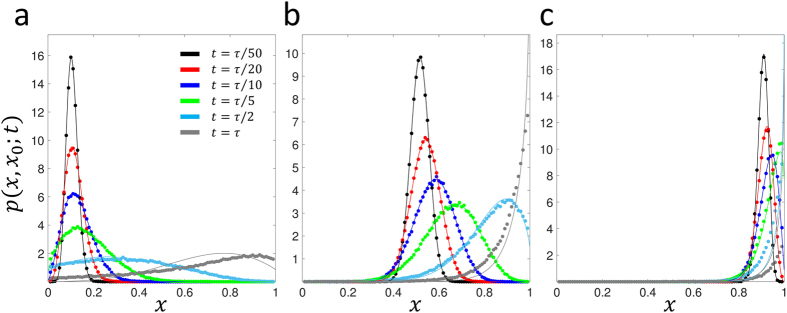
Comparison of approximate calculation of the TPDF for drift and selection (*Ns* = 10, *μ*_1_ = *μ*_2_ = 0, solid lines – Eqn. 11) and numerical integration of stochastic differential equation that arises from diffusion approximation (solid circles). (a) initial frequency *x*_0_ = 0.1, (b) *x*_0_ = 0.5, (c) *x*_0_ = 0.9. Green’s functions are plotted at times given by fractions of 
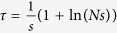
, which is approximately the expected time to fixation of a variant which survives drift and then is driven to fixation by selection^36^.

**Figure 4:**
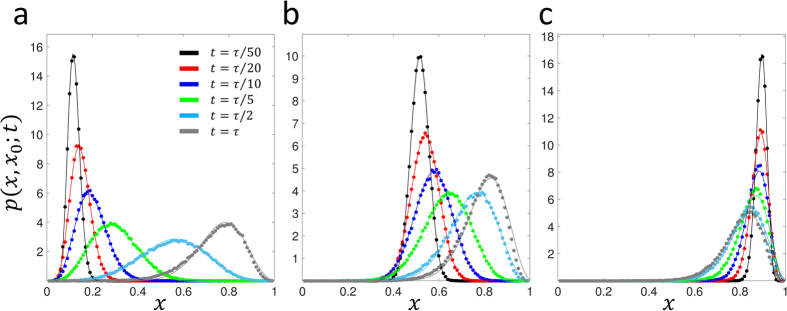
Comparison of approximate calculation of the TPDF for *Ns* = 10 and 4*Nμ* = 10, where *μ* = *μ*_1_ = *μ*_2_ (solid lines – Eqn. 11) and numerical integration of stochastic differential equation that arises from diffusion approximation (solid circles). (a) initial frequency *x*_0_ = 0.1, (b) *x*_0_ = 0.5, (c) *x*_0_ = 0.9. Green’s functions are plotted at times given by fractions of 
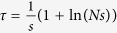
, which is approximately the expected time to fixation of a variant which survives drift and then is driven to fixation by selection^36^.

We plot the results for the neutral case *Ns* = 0 and *μ*_1_ = *μ*_2_ = 0 in Fig. 2 for *x*_0_ = 0.1 and *x*_0_ = 0.5, *Ns* = 10 and *μ*_1_ = *μ*_2_ = 0 (*x*_0_ = {0.1, 0.5, 0.9}) in Fig. 3 and *Ns* = 10 and 4*Nμ* (*μ*_1_ = *μ*_2_ = *μ*, *x*_0_ = {0.1, 0.5, 0.9}) in Fig. 4; see Supplementary Online Information for plots at wider range of selection coefficients and mutation rates, as well as at a small initial frequency *x*_0_ = {0.01, 0.99}. We find universally that for all parameter combinations the heuristic approach and the integration of the Wright-Fisher SDE (Eqn. 6) agree very well at short times compared to the average time for fixation/loss of a variant. This is true even when *x*_0_ is very close to 0 or 1, for sufficiently short times (Supplementary Online Information) and is reasonably accurate to quite long times (*t* ~ *N* or *t* ~ *τ*) for an initial frequency of *x*_0_ = 0.1 (Fig. 3A). In particular, for strong mutation and selection (Fig. 4) we see the solutions are good even at long times, since probability does not accumulate at the boundaries due to a mutation-selection balance that peaks the TPDF away from the boundaries. Note that the “deterministic” drift term in Eqn. 7 is necessary for accurate results, since otherwise, for example, for positive selection and arbitrarily small initial frequencies the mean would always increase to fixation; as Fig. 1 demonstrates, there is a critical initial frequency below which drift dominates selection and we expect the mean to decrease towards the loss boundary.

### Determination of maximum likelihood parameters

In this section we demonstrate using simulated data that the heuristic method of calculating the TPDF allows accurate determination of the population genetic parameters of interest, effective population size, selection coefficient and mutation rate, by finding the maximum of the likelihood functions. We make the assumption that the true frequencies of variants or alleles is known with perfect accuracy, whereas in practice experimental frequencies will be determined from a sampling process from the true population; this issue is dealt with by Bollback *et al*.^14^, where a hidden Markov model is used to effectively deconvolve the true frequencies from the sampled frequencies.

Given a times series of frequencies {*x*_*i*_} at times {*t*_*i*_} the likelihood of the data is given simply by


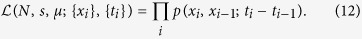


With the analytical formula for the TPDF in Eqn. 11, presented in the previous section, this can be directly evaluated very quickly.

**Figure 5:**
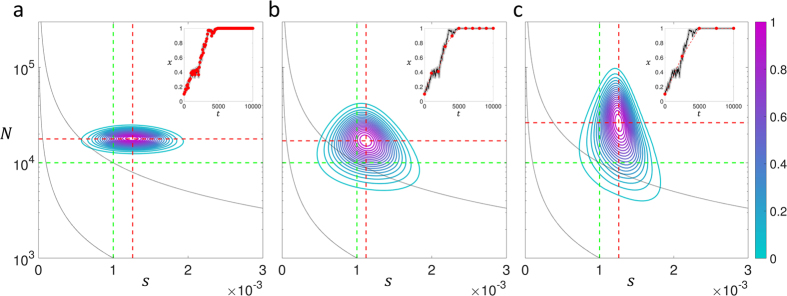
Likelihood surfaces for simulated time series, where the inset of each shows the underlying time series (black/grey line) and its sampling (red circles); (**a**) sampling time Δ*t* = 100 generations, (**b**) Δ*t* = 1000 generations, and (**c**) Δ*t* = 2500 generations. The black dotted lines represent contours of *Ns* = 1 (lower contour), which is the boundary between weak and strong selection and *Ns* = 10 (higher contour), which is the strength of selection used in the simulations. The likelihoods are scaled to their maximum value for each sampling period and contours show lines of equal likelihood, separated by values of 0.05 in likelihood, where magenta is the largest likelihood and cyan the smallest likelihood; in each case the *L* = 0.05 contour corresponds approximately to 95% of the integral of the likelihood. The green dashed lines represent the parameter values used to generate the simulated data, while the red dashed lines represent those values that maximise the likelihood; the maximum likelihood parameters are (**a**) N* = 1.8 × 10^4^, and s* = 1.3 × 10^−3^, (**b**) N* = 1.7 × 10^4^, and s* = 1.1 × 10^−3^, (**c**) N* = 2.6 × 10^4^, and s* = 1.3 × 10^−3^.

In Fig. 5, we show the likelihood surfaces for data generated using Eqn. 6 for *N* = 10000, *s* = 0.001 (*Ns* = 10) and *μ*_1_ = *μ*_2_ = 0, where the underlying data is sampled with different periods Δ*t* with a total period of *T* = 10000 generations, as shown in the insets of Fig. 5. For each sampling period, it is clear from Fig. 5 that a likelihood ratio test would reject the null hypothesis (*s* = 0) since there is no significant likelihood at the line *s* = 0; below we quantify the performance of a simple likelihood ratio test using a receiver operator characteristic (ROC) plot and compare it to the calculation of the TPDF in Feder *et al*.^17^.

**Figure 6:**
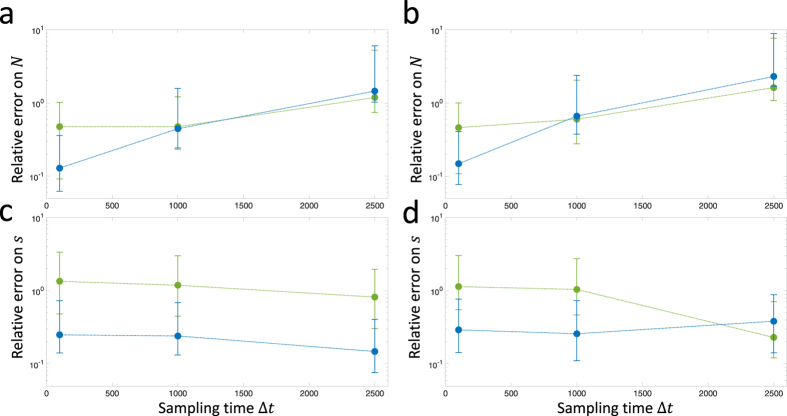
Distribution of relative error in determining effective population size *N* = 10^4^ (**a,b**) and selection coefficient *s* = 10^−3^ (**c,d**) from maximum of likelihood function, as function of sampling time, calculated over 1000 replicate simulations with initial frequency *x*_0_ = 0.1 (**a,c**) and *x*_0_ = 0.5 (**b,d**), where calculation of TPDF of current paper (blue circles) is compared to the calculation of Feder *et al*.^17^. The circles represent median values, while the error bars are the interquartile range of the distribution and not a representation of the standard error on determining the median.

However in addition, direct calculation of the likelihood function given the data means optimum parameter values can be determined. From Fig. 5, we can see that for this particular time-series, the maximum likelihood parameters are determined correctly to within an order of magnitude. In Fig. 6, we examine in more detail the accuracy of estimating the effective population size and selection coefficient from the maximum of the likelihood function, by calculating the relative error given true values of *N* = 10^4^ and *s* = 10^−3^, over 1000 replicate runs. This accuracy is compared to the calculation of the TPDF of Feder *et al*.^17^, which uses an approximation that ignores the effect of the boundary and would be expected to be inaccurate near *x* = 0 or *x* = 1; for this reason, we make the comparison for initial frequencies of *x* = 0.1 (Fig. 6a,c) and *x*_0_ = 0.5 (Fig. 6b,d). The calculation of Feder *et al*., is based on the Moran model and so to compare to simulations using the Wright-Fisher model, times in the Moran model need to be scaled by a half. We find that overall the calculation in this paper significantly outperforms the calculation of Feder *et al*.^17^ in determining the selection coefficient, with a median relative error of 10% to 30% for *x*_0_ = 0.1 and 30% to 40% for *x*_0_ = 0.5 compared to approximately 100% for the Feder calculation; the exception is for long sampling times (Δ*t* = 2500) and *x*_0_ = 0.5, where the Feder calculation slightly outperforms the calculation in this paper, although it is not clear why at longer sampling times the Feder calculation should do better compared to shorter sampling times. Interestingly, the results suggest that the selection coefficient can be determined more accurately from time-series that sweep to fixation from a smaller initial frequency compared to one that starts with an intermediate frequency. In estimating the effective population size, the current calculation outperforms the Feder calculation for frequent sampling (Δ*t* = 100, with a median relative error ≈12% versus ≈40–50%), but performs similarly for the longer sampling times. That the effective population size is less accurately determined, compared to the selection coefficient, as the sampling time Δ*t* increases is easily understood since the selection coefficient is mainly determined from the deterministic changes in variant frequency, whilst the effective population size from deviations from this deterministic behaviour, caused by the spreading of the TPDF; increasing Δ*t* means that there is less accurate sampling of these fluctuations and the accuracy of the determined effective population size decreases. Finally for reference, Δ*t* = 1000 and Δ*t* = 2500 correspond to a frequency of times points (relative to the timescale of change in frequency) similar to previous experimental time-series^14^,^16^.

**Figure 7:**
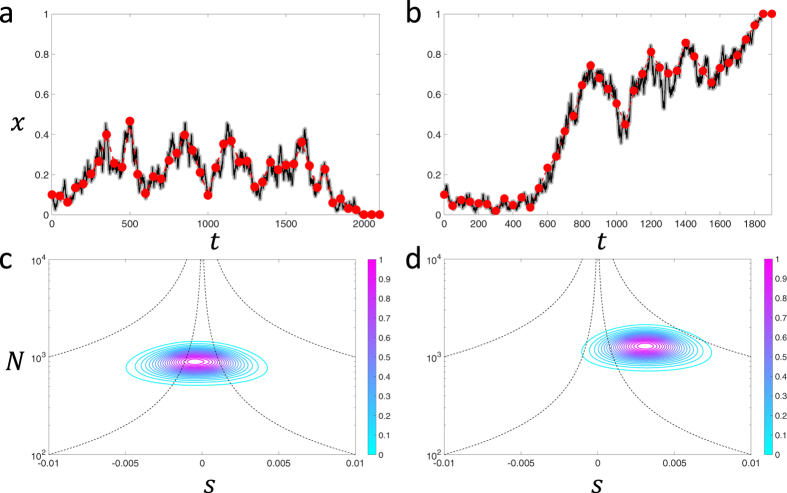
Likelihood surfaces for simulated neutral time series (*N* = 1000, *s* = 0, *μ* = 0) as shown by grey/black lines in (**a,b**), and sampled every 50 generations, indicated by red filled circles. The dotted lines represent contours of *N*|*s*| = 1 (lower contour) and *N*|*s*| = 10 (higher contour). In (**c**) we have the likelihood surface for the time series shown in (**a**) and we see that the likelihood is approximately centred around a selection coefficient of zero, given the width of the likelihood function; on the other hand in (**d**) we have the likelihood surface for the time series in (**b**), which has a clear shift to positive selection coefficients.

**Figure 8:**
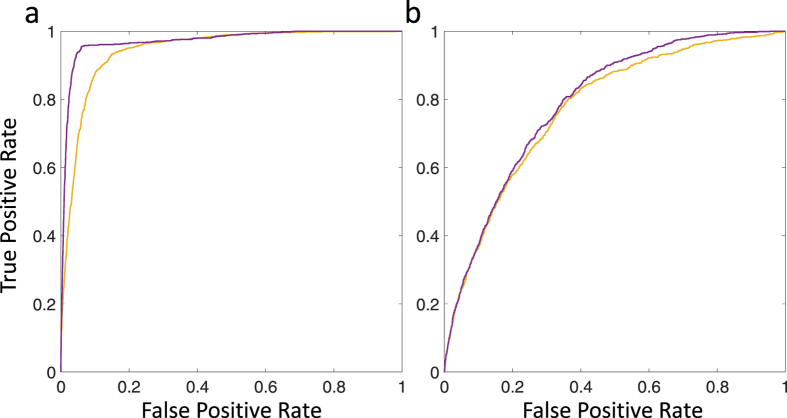
Receiver operator characteristic (ROC) curve for 10000 sites with 10% of sites with a selection coefficient of *s* = 10^−3^ with the rest neutral (*s* = 0), for an effective population size of *N* = 10^4^ (*Ns* = 10). (**a**) shows the ROC plot for *x*_0_ = 0.1 and (**b**) for *x*_0_ = 0.5, where the purple line is based on the TPDF calculated in this paper and the yellow line based on the calculation in Feder *et al*.^17^.

We now examine the case where time series are purely due to neutral drift. In Fig. 7, we show two sample trajectories for the case that *N* = 1000, *s* = *μ* = 0 and the resulting likelihood functions. For the trajectory shown in Fig. 7a with likelihood in Fig. 7c, a likelihood ratio test would decide in favour of the neutral hypothesis. However, there is some probability that by chance, such as in Fig. 7b with likelihood in Fig. 7d, trajectories will arise that indicate the presence of positive selection. This multiple comparison problem is common in statistical testing and standard multiple hypothesis methods can be used^37^, for example as in the field of molecular evolution, where the family-wise false positive rate is controlled across all tested sites in a genome^38^,^39^. Here, to assess how well the current calculation of the TPDF performs in rejecting the null hypothesis (*s* = 0) compared to the calculation of Feder *et al*.^17^, we plot in Fig. 8 the receiver operator characteristic (ROC) curve for the case where there are 10000 sites to be tested (for example, 10000 nucleotides in a genome) using a likelihood ratio test (LRT), with 10% of sites with a selection coefficient of *s* = 10^−3^ with the rest neutral (*s* = 0), where the effective population size is *N* = 10^4^ (*Ns* = 10). Here, we use the LRT as a simple test statistic to assess relative performance, although as shown by Feder *et al*., the LRT is a biased statistic to reject the null-hypothesis. The ROC plot is produced by taking the LRT test statistics across all sites and ordering them by how strongly they reject the null hypothesis (favour selection) and then plots the rate of true positives vs false positives (*TPR* vs *FPR*) in the ordered list; a perfect ROC curve would have a vertical line from the origin to *TPR* = 1, which represents all sites under selection being detected correctly, followed by a horizontal line to *FPR* = 1 represent all the sites evolving neutrally. The ROC plots show that the calculation of the TPDF in the current paper is more sensitive to detecting selection than the Feder calculation, for *x*_0_ = 0.1, as it rises more steeply initially with a small number of false positives amongst the true positives. On the other hand for *x*_0_ = 0.5 both calculations perform worse compared to *x*_0_ = 0.1 with only a marginal improvement over the Feder calculation in the sensitivity of detecting selection. This is as expected as the Feder calculation is expected to be only accurate away from the boundaries (*x* ≠ 0 and *x* ≠ 1). In addition, as seen in Fig. 6, time-series of variant frequencies under selection that start with a smaller frequency can be detected more easily.

**Figure 9:**
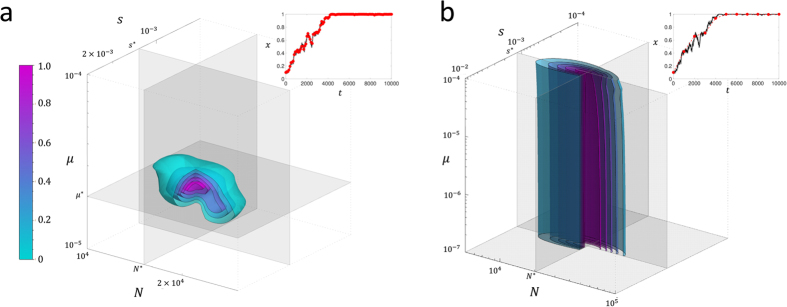
Likelihood contour surfaces for simulated time series for parameter values, *N* = 10000, *s* = 0.001 and *μ* = *μ*_1_ = *μ*_2_ = 10^−5^ (4*Nμ* = 0.4), as shown by grey/black line in inset of each figure: (**a**) sampling time Δ*t* = 100 generations (red circles in inset) and b) Δ = 1000 generations (red circles in inset). The likelihood is scaled to its maximum value and contours show surfaces of equal likelihood, where magenta is the largest likelihood and cyan the smallest likelihood. The grey orthogonal planes are positioned at the parameter values that maximise the likelihood and the cut-away corresponds to the *N* − *μ* and *s* − *μ* planes; the maximum likelihood parameters are (**a**) N* = 1.51 × 10^4^, s* = 1.3 × 10^−3^ and μ* = 2 × 10^−5^ and (**b**) N* = 2.04 × 10^4^, s* = 1.2 × 10^−3^ and μ* is undefined.

The heuristic solution for the TPDF (Eqn. 11) also allows the mutation rate to be a parameter. However, typically the most likely scenario when there are only two variants in the population is that mutation is weak; it is therefore interesting to examine whether mutation rate can be determined in this case, particularly as mutations will tend to have strong effects only near the boundaries. In Fig. 9, we show the likelihood surface contours for data generated using Eqn. 6 (shown in inset of Fig. 9) for *N* = 10000, *s* = 0.001 (*Ns* = 10) and *μ* = *μ*_1_ = *μ*_2_ = 10^−5^ (4*Nμ* = 0.4). For a short sampling period of Δ*t* = 100, we see that all the parameters are determined with reasonable or good accuracy; the effective population size is determined with an error of 4.5%, the selection coefficient 4% and the mutation rate 6%, on a log scale, which shows they are all estimated to the correct order of magnitude. However, if we increase the sampling time to Δ*t* = 1000, while the error on the population size and selection coefficient are similar, we see from Fig. 9 that the likelihood function becomes almost invariant with respect to mutation rate, making the optimum undetermined. This arises since the longer sampling period misses the fluctuations away from fixation, which provide information about the mutation rate, as seen in the time series, shown in the inset of Fig. 9a. So as one would expect the sampling period needs to be shorter than the inverse of the rate at which mutations enter the population, *Nμ*; even at Δ*t* = 100 generations the sampling of these fluctuations is quite poor, however, still sufficient to allow a reasonable determination of the mutation rate.

**Figure 10:**
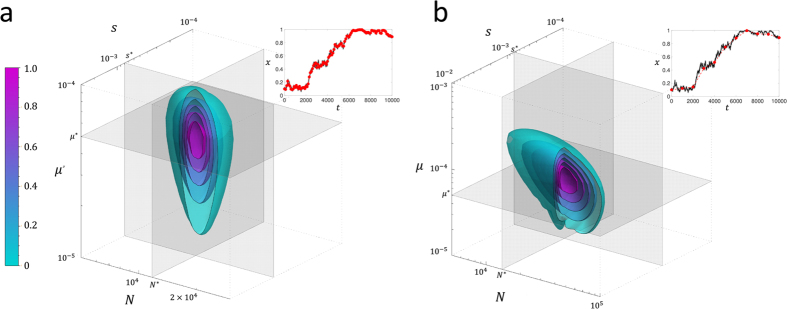
Likelihood contour surface for simulated time series for parameter values, *N* = 10000, *s* = 0.001 and *μ* = *μ*_1_ = *μ*_2_ = 10^−4^ (4*Nμ* = 4), as shown by grey/black line in inset of each figure: (**a**) sampling time Δ*t* = 100 generations (red circles in inset) and (**b**) Δ = 1000 generations (red circles in inset). The likelihood is scaled to its maximum value and contours show surfaces of equal likelihood, where magenta is the largest likelihood and cyan the smallest likelihood. The grey orthogonal planes are positioned at the parameter values that maximise the likelihood and the cut-away corresponds to the *N* − *μ* and *s* − *μ* planes; the maximum likelihood parameters are (**a**) N^*^ = 1.17 × 10^4^, s^*^ = 7.94 × 10^−4^ and μ^*^ = 5.01 × 10^−5^ and (**b**) N^*^ = 1.38 × 10^4^, s^*^ = 7.59 × 10^−4^ and μ^*^ = 5.01 × 10^−5^.

On the other hand when mutation is strong, as shown in Fig. 10a, we find for a short sampling period of Δ*t* = 100 a similar accuracy in determining *N*, *s*, *μ*. As we increase the sampling period to Δ*t* = 1000, as shown in Fig. 10b, we find that the mutation rate can still be determined within an order of magnitude, unlike when mutations are weak.

## Discussion

Despite, being known for almost a century, Fisher’s angular transformation, has received little attention. Under the transformation, the stochastic dynamics of neutral, or genetic drift, which is characterised by a co-ordinate dependent diffusion constant, can be transformed to simple, co-ordinate independent, Brownian motion. Intuitively, this transforms the co-ordinate system to one where equal mean square distances are traversed in equal times giving co-ordinate independent diffusion. The result, however, is an effective unstable convective potential or force, that drives trajectories to fixation or loss; as we show explicitly this convective potential represents the *flux* of diffusers to the boundaries in *x*–space, but not the convection, as it is simple to demonstrate there is no net direction or convection of individual trajectories in *x*–space. This is an example of flux without convection, as discussed by Lancon *et al*.^27^, but highlighted here for the first time in the context of population dynamics.

A possible reason for Fisher’s angular transformation remaining an intellectual curiosity, is that the resultant convective force is non-linear and with the addition of selection becomes particularly complicated. However, we show for the first time that within the transformed space very accurate approximations of the 2-allele transition probability density of population genetics for arbitrary selection coefficient, population size and mutation rates between variants can be calculated. This is achieved here by introduction of a heuristic technique, similar to previous approaches^17^,^33^,^34^,^35^, that assumes a Gaussian distribution whose mean follows a solution to the infinite *N* or deterministic equation of motion and variance slaved to the local curvature evaluated from the mean solution. Together with the heuristic Gaussian approximation this represents, to the author’s knowledge, a novel general approach for asymptotically solving Fokker-Planck equation’s with a co-ordinate dependent diffusion constant, which result in slowly-varying potentials (or equivalently SDEs with multiplicative noise), where the solution to the mean behaviour is known; indeed, in 1-dimension a PDE with co-ordinate dependent diffusion can always be transformed to one with co-ordinate independent diffusion^26^,^40^. For more than two variants and interactions between loci, including recombination and linkage, the methods detailed in ref. 26, suggests via the metric tensor, a potential route to finding solutions for the transition probability function. The generality of the heuristic Gaussian solution means that a whole host of different and previously intractable evolutionary problems could be addressed, including for example, frequency-dependent and fluctuating selection.

Finally, we show that these solutions of the Wright-Fisher process can sensitively detect selection and in principle lead to accurate determination of parameter values from simulated data, given sufficiently frequent sampling of the underlying time-series of variant change and strong selection. As real population variant/allele frequencies are sampled from a larger underlying population, methods for example used in ref. 14 using a hidden Markov model for the true frequency of variants, would be required for an accurate statistical test.

These results have application to detecting selection in time-series data of the composition of variants, in biological evolution, language evolution and for species in ecosystems. In particular, as these results have particular accuracy in the asymptotic short-time limit, they will be applicable to studying selection from time-series of variants (haplotypes) in virus evolution, and potentially for single genomic sites in viruses with strong recombination, such as HIV, since they have large effective population sizes and short generation times, meaning even sampling virus populations infrequently (on the time scale of many months or years) would be accurately modelled by the results of this paper.

## Methods

### Boundary conditions

As mentioned in the main text the solution presented by the Gaussian heuristic method does not obey the boundary conditions at *x* = 0 or *x* = 1, which for zero mutation rate, require the solution to be non-zero and finite at these boundary points, so that there is a non-zero flux to the fixation or loss boundaries (see ref. 41 for a detailed discussion); in the angular space this translates to effective absorbing boundary conditions, where specifically for *θ* ≪ 1 we require *q*(*θ*, *θ*_0_; *t*) ~ *θ*, since the Jacobian 
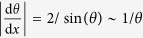
. A similar argument means that we need *q*(*θ*; *θ*_0_; *t*) ~ *π* − *θ* for *π* − *θ* ≪ 1. As Eqn. 11 does not obey these boundary conditions it develops singularities at the boundary for long times compared to the time for fixation. To counter this tendency for long times, we multiply the solution in angular space by a weighting function 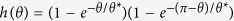, which has the property that for *θ* ≪ θ^*^, *h*(*θ*) ~ *θ* and for (*π* − *θ*) ≪ θ^*^, *h*(*θ*) ~ *π* − *θ* and for other regions away from the boundary *h*(*θ*) ≈ 1. We choose a sufficiently small value for θ^*^ dependent on the strength of selection *Ns* and the initial frequency *x*_0_; when selection is weak on the initial variant, i.e. 4*Nsu* ≪ 1, where *u* = *x*_0_ or *u* = 1 − *x*_0_, then we want to ensure that 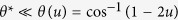 and so we choose 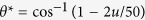; in the converse case, where selection is strong (4*Nsu* ≫ 1), we choose a fixed θ^*^ = 0.1, since in this case, if for example, *x*_0_ is close to 1 and *s* > 0, then selection tends to build up density near the boundary more quickly than in the neutral case and so a larger θ^*^ is required. For intermediate values of the strength of selection on the initial variant we interpolate between these two values using a tanh switching function centred on zero and width 0.5 with respect to the parameter ln(4*Nsu*). For the case of a non-zero mutation rate, the boundary conditions are zero flux (*J*(0) = *J*(1) = 0) at the boundaries and we do not include this weighting function; as can be seen from Eqn. 11, this means that the TPDF diverges for the transition between any finite frequency to *x* = 0 or *x* = 1 and so we remedy this with a pragmatic approach, where occurrences in the data of *x* = 0 or *x* = 1 are transformed to 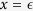 and 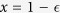, where we choose 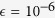, an arbitrarily small number.

Finally, instead of using the full form of the effective convective force in the theta domain, we expand the terms that diverges as *θ* → {0, *π*} to third order to give finite and well-behaved derivatives at and near the boundaries, so that the derivative of Eqn. 5 becomes
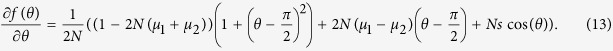
This is reasonable since the solution in any case only approximately obeys the boundary conditions and this ensures that the variance remains well-behaved as the mean approaches the boundary.

## Additional Information

**How to cite this article**: Khatri, B. S. Quantifying evolutionary dynamics from variant-frequency time series. *Sci. Rep.* **6**, 32497; doi: 10.1038/srep32497 (2016).

## Supplementary Material

Supplementary Information
