# Supplementary material for: Quantifying evolutionary dynamics from variant-frequency time series
Source: Sci Rep. 2016 Sep 12;6:32497. doi: 10.1038/srep32497 (PMC5018853; doi:10.1038/srep32497)
Supplement: Supplementary Information [file srep32497-s1.pdf]

Supplementary Information:

Quantifying evolutionary dynamics from variant-frequency time series

HARMONIC APPROXIMATION FOR NEUTRAL TRANSITION PROBABILITY DENSITY FUNCTION

The neutral Fokker-Planck equation in angular space (Eqn.3 in the main text) has a representation as an Ito stochastic differential equation[1,2]

$$\frac{\mathrm{d}\theta}{\mathrm{d}t} = -\frac{1}{2N}\cot(\theta) + \eta(t), \tag{1}$$

where $\langle \eta(t) \rangle = 0$ and $\langle \eta(t)\eta(t') \rangle = \delta(t-t')/N$. To calculate an approximate solution of the TPDF, we make a Taylor expansion of the force about the fixed point $\theta = \pi/2$ to linear order to give a linear stochastic differential equation,

$$\frac{\mathrm{d}\theta}{\mathrm{d}t} = \frac{1}{2N}\left(\theta - \frac{\pi}{2}\right) + \eta(t), \tag{2}$$

As we will see this approximation works well even for initial frequencies of order 10% ($x(0) = 0.1$), due to the non-linearity of the angular transformation, which has the property of compressing the central range in $x-$space about $x = 1/2$ to a smaller central region in $\theta-$ space about $\theta = \pi/2$ (as seen in Fig.1 of the main text, where the top axis are various values of x, where for example, $x = 0.1 \to \theta = 0.64$ and $x = 0.9 \to \theta = 2.5$). Equivalently, this is an harmonic approximation of the effective potential function in $\theta-$space, where $\dot{\theta} = \partial_\theta U(\theta) + \eta(t)$ and $U(\theta) = \frac{1}{2N}\ln\sin\theta$. As the resulting SDE is linear the solution is straightforwardly computed as

$$\theta(t) = \frac{\pi}{2} + \left(\theta_0 - \frac{\pi}{2}\right)e^{t/2N} + \int_0^t \mathrm{d}t'\eta(t')e^{\frac{t-t'}{2N}}, \tag{3}$$

where $\theta_0 = \theta(0)$. As the solution is a sum of Gaussian random variables η the TPDF for θ will be Gaussian with mean,

$$\langle \theta(t) \rangle = \frac{\pi}{2} + \left(\theta_0 - \frac{\pi}{2}\right)e^{t/2N}, \tag{4}$$

since $\langle \eta(t) \rangle = 0$ and variance,

$$\langle\langle \theta^2(t) \rangle\rangle = e^{t/N} - 1, \tag{5}$$

where the van Kampen notation has been used, $\langle\langle \theta^2 \rangle\rangle = \langle \theta^2 \rangle - \langle \theta \rangle^2$. Note that the variance diverges for $t \gg N$ and the mean divergences for $t \gg 2N$, to $-\infty$ when $\theta_0 < \pi/2$ and to $+\infty$ for $\theta_0 > \pi/2$ and is fixed for all time at $\langle \theta \rangle = \pi/2$, if $\theta_0 = \pi/2$ the fixed point of the effective drift dynamics. The TPDF function in $\theta-$space is then

$$q(\theta, \theta_0; t) = \frac{1}{\sqrt{2\pi(e^{t/N}-1)}}\exp\left(-\frac{\left(\theta - \theta_0 e^{t/2N} - \frac{\pi}{2}(1-e^{t/2N})\right)^2}{2(e^{t/N}-1)}\right). \tag{6}$$

Note the similarity of form to the TPDF of an overdamped harmonic oscillator, but with the difference that, as discussed, here the mean and variance diverge[3]. This solution does not obey the boundary conditions at $\theta = 0$ and $\theta = \pi$, which are required to be absorbing and specifically to go linearly to zero at these points; this is in order for the solution in $x-$space to be finite at the boundaries, as required due to the singularity of the diffusion constant at $x = 0$ and $x = 1$[4]. The method of images cannot be used in this case as the required image has it's forces reversed and so does not obey the original Fokker-Planck equation. Transforming back to $x-$space, we have,

$$p_x(x, x_0; t) = \left|\frac{\mathrm{d}\theta}{\mathrm{d}x}\right| q(\theta(x), \theta_0(x_0); t)$$
$$= \frac{1}{\sqrt{2\pi x(1-x)(e^{t/N}-1)}} \exp\left(-\frac{\left(\cos^{-1}(1-2x) - \cos^{-1}(1-2x_0)e^{t/2N} - \frac{\pi}{2}(1-e^{t/2N})\right)^2}{2\left(e^{t/N}-1\right)}\right), \qquad (7)$$

where the Jacobian is $\left|\frac{\mathrm{d}\theta}{\mathrm{d}x}\right| = 2/\sin\theta = 1/\sqrt{x(1-x)}$.

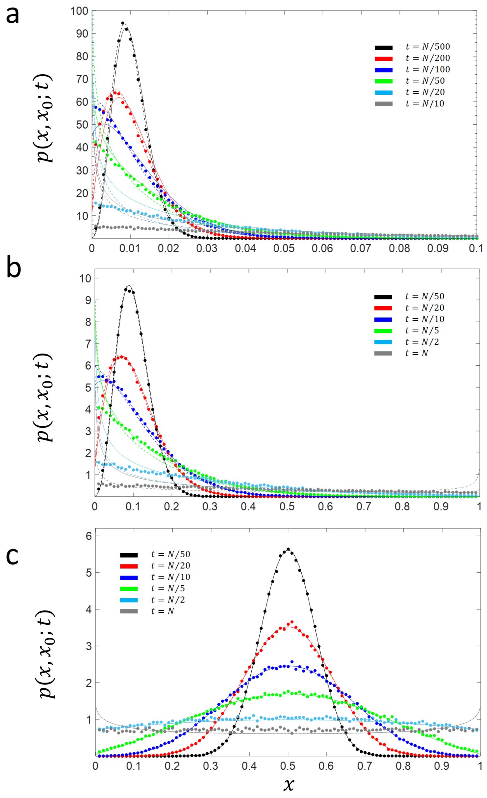

FIG. 1. Comparison of approximate calculation of neutral TPDF (solid lines – Eqn.7 and for comparison the heuristic approximation of the main text in dashed lines) and numerical integration of stochastic differential equation that arises from diffusion approximation (solid circles). a) initial frequency $x_0 = 0.01$, b) $x_0 = 0.1$, c) $x_0 = 0.5$.

The results are plotted in Fig.1, at various times and initial conditions, as solid lines and compared against numerical integration of the exact neutral Wright-Fisher stochastic differential equation (Eqn.1). We see that, in general, the approximation works very well for short times $t \ll N$ and when the initial frequency x_0 is not too small. More precisely we would expect the approximation to be good for sufficiently short times compared to the average time to fixation, which is $\langle t^* \rangle = -2N(x_0 \ln(x_0) + (1-x_0)\ln(1-x_0))$; for $x_0 = \{0.01, 0.1, 0.5\}$, $\langle t^* \rangle \approx \{0.1N, 0.7N, 1.4N\}$, which is consistent with the results in Fig.1. The solution has a simplicity, where it is clear the behaviour of gene frequencies is essentially that of Brownian motion in an unstable harmonic potential; the non-linearity in $x-$space arises purely from working in the more natural co-ordinates of the angular transformation, where in particular the argument of the exponential in the Gaussian solution is just the square of the stochastic distance between x_0 and x. However, we see that the heuristic Gaussian approximation of the main text (dashed lines) is in general more accurate. Finally, as shown in the main text, the inclusion of mutation, where $\mu = \mu_1 = \mu_2$ gives the same stochastic dynamics under Fisher's angular transformation, but with the rescaling of the effective potential with coefficient $-\frac{1}{2N} \rightarrow 2\mu - \frac{1}{2N}$, so the results here are applicable to neutral drift dynamics including two-way equal mutation; it is clear that when $4N\mu > 1$ the solutions will be exactly that of an over-damped harmonic oscillator in θ-space.

DETAILED COMPARISON OF HEURISTIC CALCULATION OF TPDF VS LANGEVIN SIMULATIONS

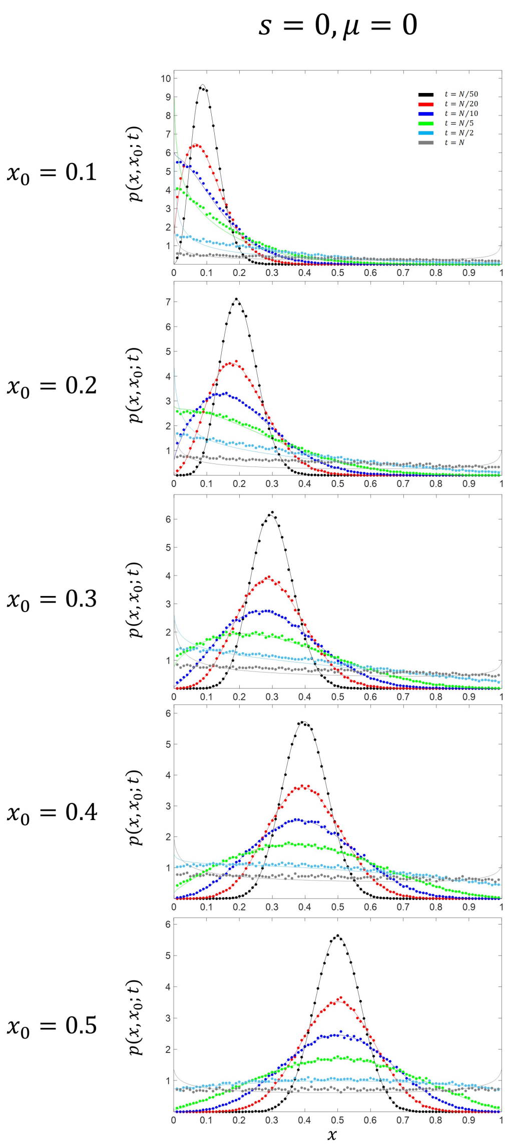

FIG. 2. Comparison of heuristic Gaussian approximation of neutral TPDF (solid lines) and numerical integration of stochastic differential equation that arises from diffusion approximation (solid circles), for various initial frequencies as shown on left of each plot.

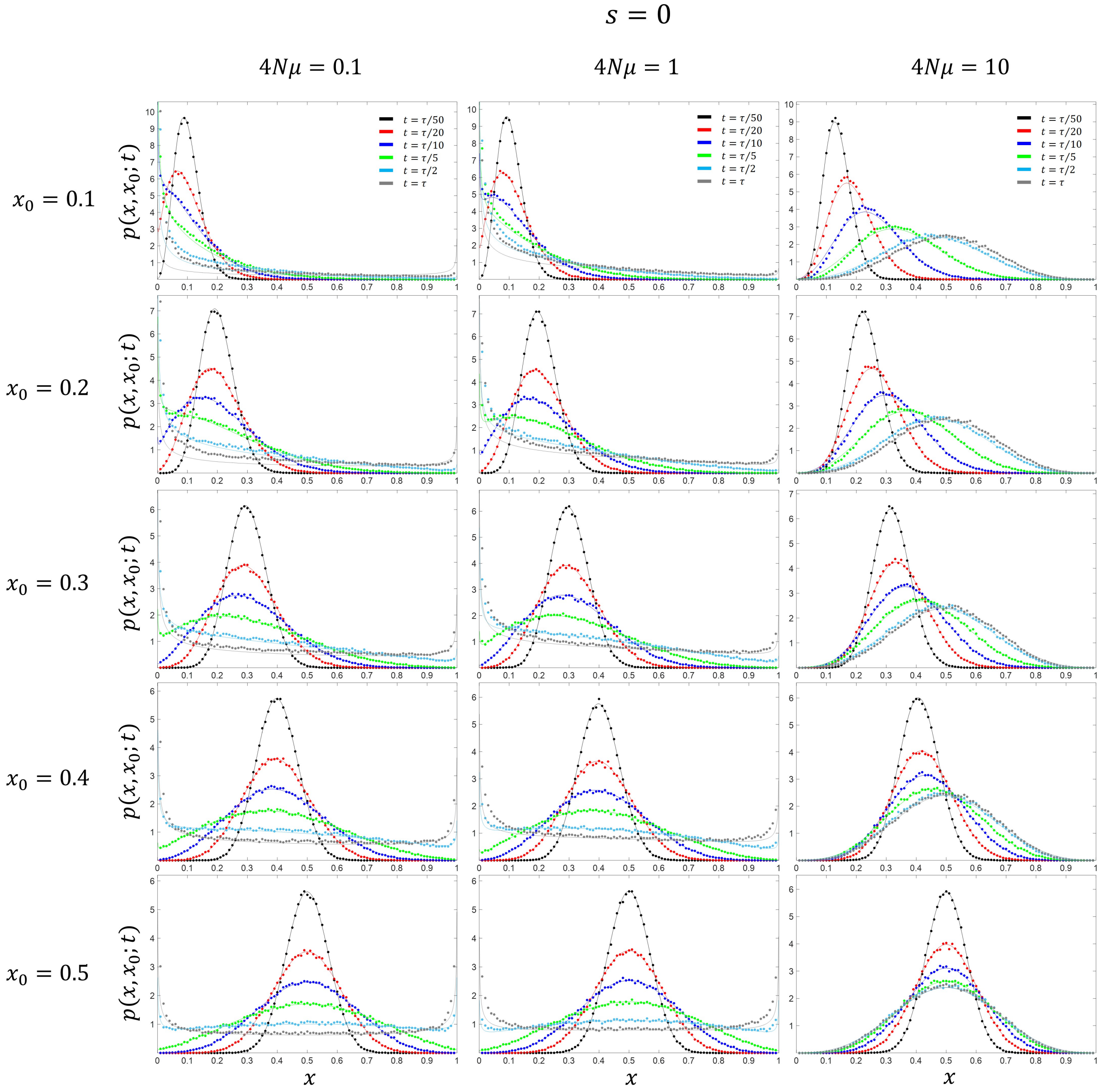

FIG. 3. Comparison of heuristic Gaussian approximation of TPDF for drift and mutation ($s = 0$), where $\mu = \mu_1 = \mu_2$, (solid lines) and numerical integration of stochastic differential equation that arises from diffusion approximation (solid circles), for various initial frequencies as shown on left of each plot and strength of mutation varied, as shown on the top. We see that going from $4N\mu = 0.1$ to $4N\mu = 1$ only changes the TPDF little, but there is a significant change when $4N\mu = 10$, where strong mutations lead to a stable intermediate polymoprhism/variant frequency. Times for each plot are fractions of $\tau = \frac{1}{s}(1 + \ln(Ns))$, which is approximately the expected time to fixation of a mutant which survives drift and then is driven to fixation by selection[5].

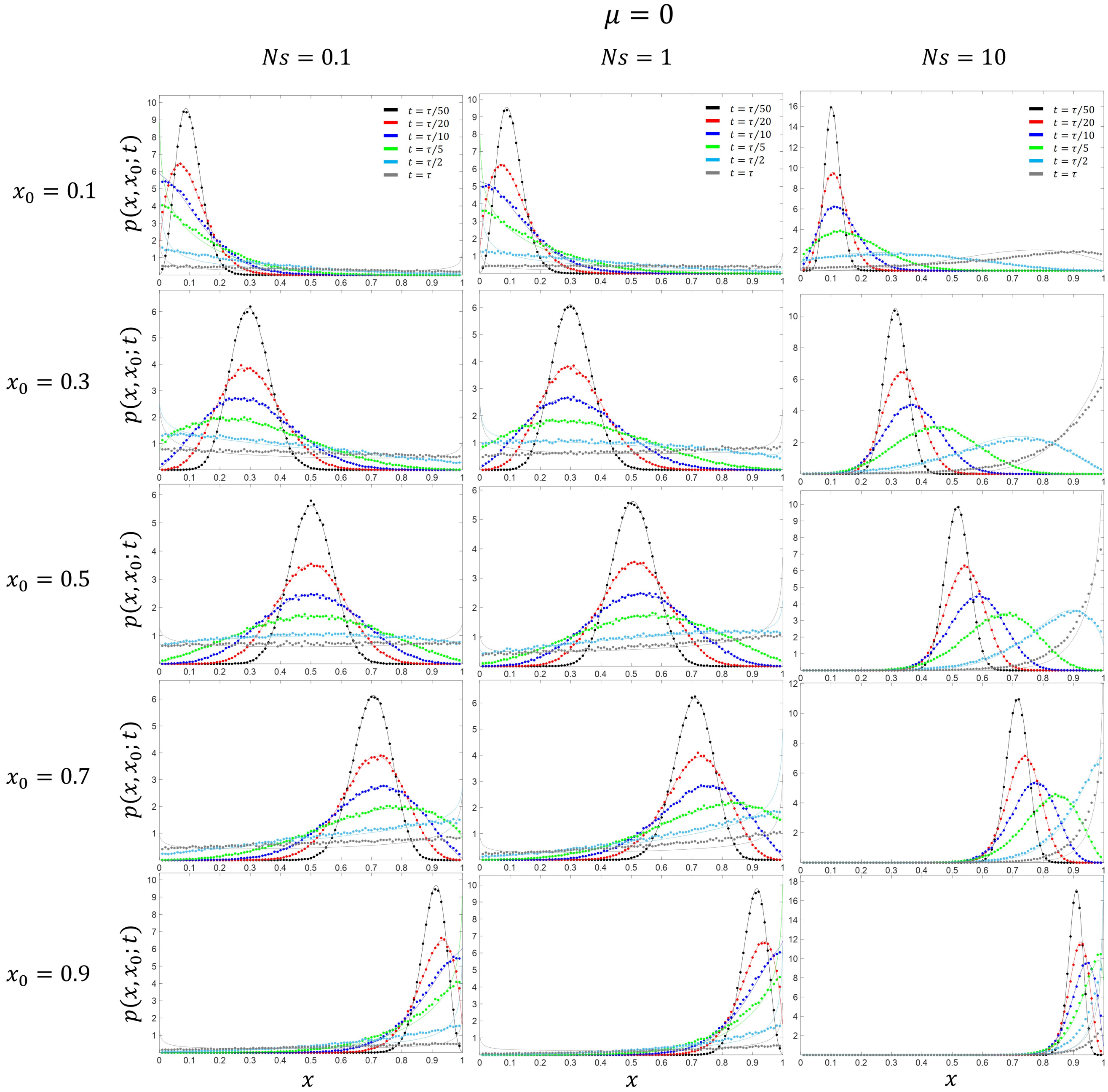

FIG. 4. Comparison of heuristic Gaussian approximation of TPDF for drift and selection, where $\mu = \mu_1 = \mu_2 = 0$, (solid lines) and numerical integration of stochastic differential equation that arises from diffusion approximation (solid circles), for various initial frequencies as shown on left of each plot and strength of selection varied, as shown on the top. Times for each plot are fractions of $\tau = \frac{1}{s}(1 + \ln(Ns))$, which is approximately the expected time to fixation of a mutant which survives drift and then is driven to fixation by selection[5].

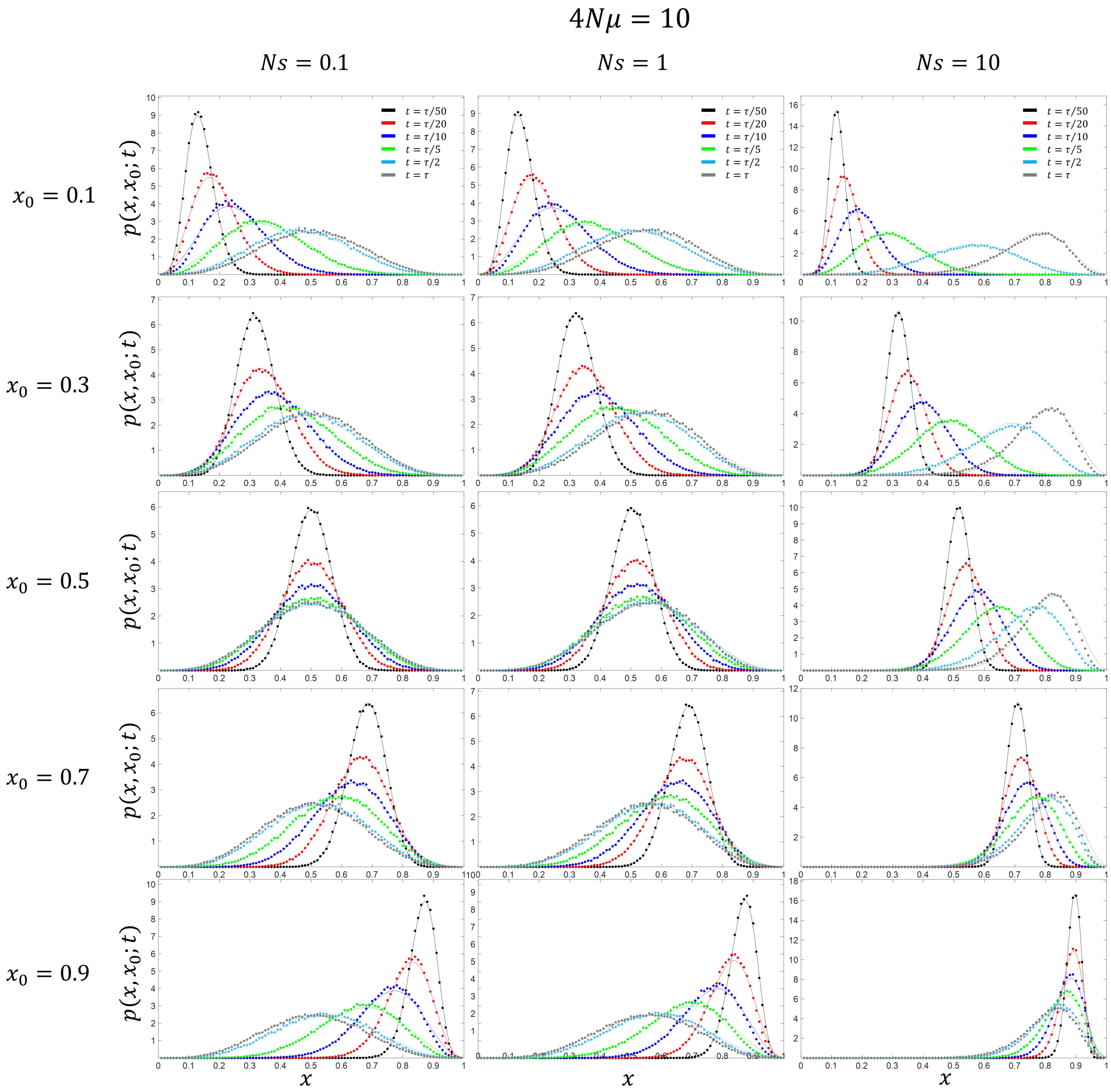

FIG. 5. Comparison of heuristic Gaussian approximation of TPDF for drift, selection, and strong mutation, where $\mu = \mu_1 = \mu_2$ and $4N\mu = 10$, (solid lines) and numerical integration of stochastic differential equation that arises from diffusion approximation (solid circles), for various initial frequencies as shown on left of each plot and strength of selection varied, as shown on the top. Times for each plot are fractions of $\tau = \frac{1}{s}(1 + \ln(Ns))$, which is approximately the expected time to fixation of a mutant which survives drift and then is driven to fixation by selection[5].

COMPARISON OF HEURISTIC CALCULATION OF TPDF VS LANGEVIN SIMULATIONS FOR SMALL INITIAL FREQUENCIES AND TIMES

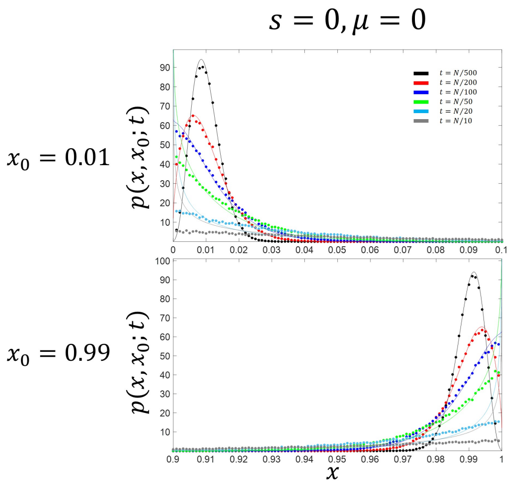

FIG. 6. Comparison of heuristic Gaussian approximation of neutral TPDF (solid lines) and numerical integration of stochastic differential equation that arises from diffusion approximation (solid circles), for small initial frequencies as shown on left of each plot. Note that the times for which the TPDF is examined is 10 times smaller than in the previous sections, ranging from $t = N/500$ to $t = N/10$. We see that in general that the form of the pdfs are similar to when $x_0 = 0.1$ but on the shorter timescale, which as we would expect as the time to fixation $\approx -2Nx_0 \ln(x_0)$ which has a weak logarithmic deviation from linear behaviour.

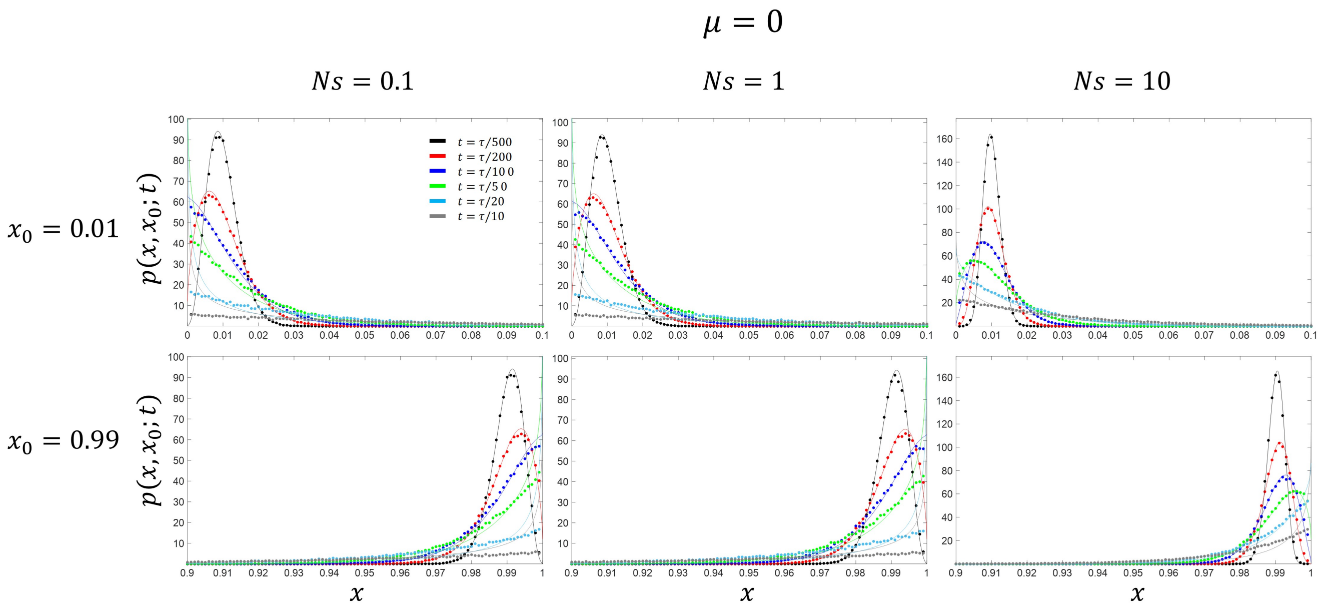

FIG. 7. Comparison of heuristic Gaussian approximation of TPDF for drift and selection, where $\mu = \mu_1 = \mu_2 = 0$, (solid lines) and numerical integration of stochastic differential equation that arises from diffusion approximation (solid circles), for small initial frequencies as shown on left of each plot and strength of selection varied, as shown on the top. Times for each plot are fractions of $\tau = \frac{1}{s}(1 + \ln(Ns))$, which is approximately the expected time to fixation of a mutant which survives drift and then is driven to fixation by selection[5]. Note that the times for which the TPDF is examined is 10 times smaller than in the previous sections, ranging from $t = \tau/500$ to $t = \tau/10$.

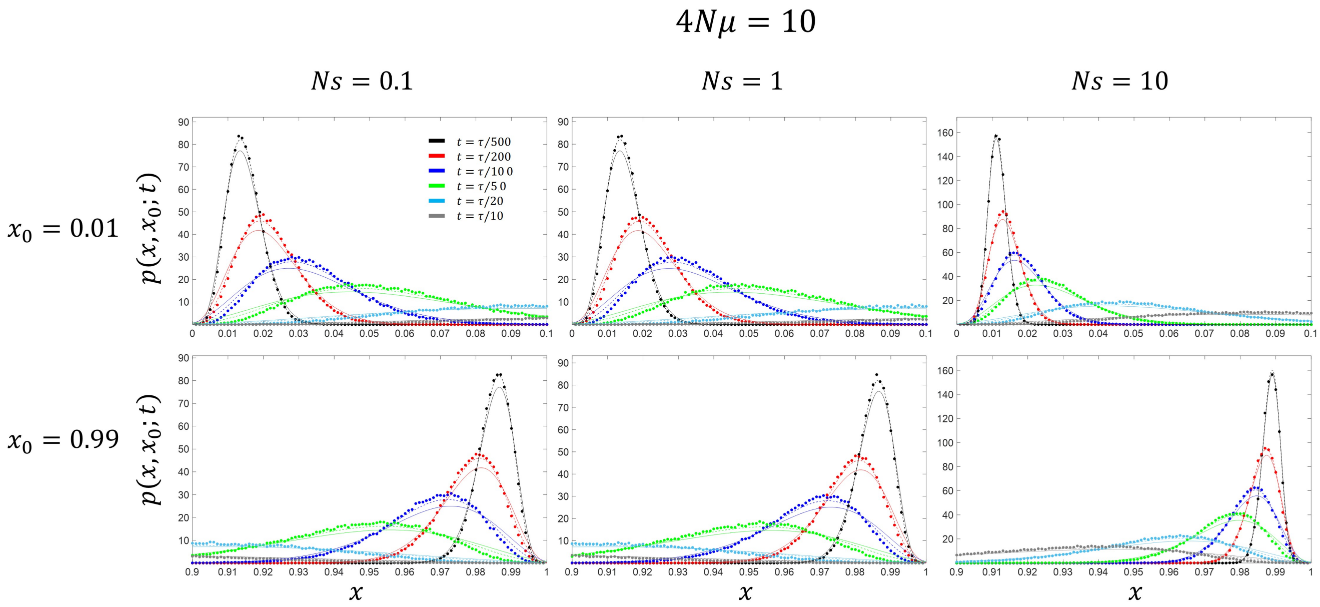

FIG. 8. Comparison of heuristic Gaussian approximation of TPDF for drift, selection, and strong mutation, where $\mu = \mu_1 = \mu_2$ and $4N\mu = 10$, (solid lines) and numerical integration of stochastic differential equation that arises from diffusion approximation (solid circles), for small initial frequencies as shown on left of each plot and strength of selection varied, as shown on the top. In dashed lines are plotted the heuristic approximation but using the full form of the convective force (Eqn.5 in the main text); we see that the full form gives a more accurate approximation than the polynomial approximation of the convective force and so in general would be more preferable when the mutation rate is strong ($4N\mu \gg 1$) – however, in a maximum likelihood application in order to keep the solution nestable over all parameters and for potentially infrequent sampling, the polynomial approximation is seen to give a good representation of the true TPDF. Times for each plot are fractions of $\tau = \frac{1}{s}(1 + \ln(Ns))$, which is approximately the expected time to fixation of a mutant which survives drift and then is driven to fixation by selection[5]. Note that the times for which the TPDF is examined is 10 times smaller than in the previous sections, ranging from $t = \tau/500$ to $t = \tau/10$.

[1] Gardiner, C. *Stochastic Methods: A Handbook for the Natural and Social Sciences* (Springer, 2009).

[2] van Kampen, N. *Stochastic Processes in Physics and Chemistry* (North-Holland, 1981).

[3] Doi, M. & Edwards, S. *The Theory of Polymer Dynamics* (Oxford University Press, 1986).

[4] Baxter, G. J., Blythe, R. A. & McKane, A. J. Exact solution of the multi-allelic diffusion model. *Math Biosci* **209**, 124–170 (2007). URL http://dx.doi.org/10.1016/j.mbs.2007.01.001.

[5] Desai, M. M. & Fisher, D. S. Beneficial mutation selection balance and the effect of linkage on positive selection. *Genetics* **176**, 1759–1798 (2007). URL http://dx.doi.org/10.1534/genetics.106.067678.
